# 2-Amino­anilinium 2-carb­oxy­acetate

**DOI:** 10.1107/S160053681102544X

**Published:** 2011-07-02

**Authors:** Li-ping Feng, Liang Zhao

**Affiliations:** a Department of Chemical & Environmental Engineering, Anyang Institute of Technology, Anyang 455000, People’s Republic of China

## Abstract

In the crystal structure of the title compound, C_6_H_9_N_2_^+^·C_3_H_3_O_4_^−^, all the amino H atoms are involved in inter­molecular N—H⋯O hydrogen bonds, which link the ions into double chains parallel to [101]. In the anion, an intra­molecular O—H⋯O hydrogen bond is observed.

## Related literature

For background to pharmaceutical applications and growth of co-crystals, see: Almarsson & Zaworotko (2004 ▶); Blagden *et al.* (2008 ▶); Vishweshwar *et al.* (2006 ▶); Kapildev *et al.* (2011 ▶); Schultheiss & Newman (2009 ▶).
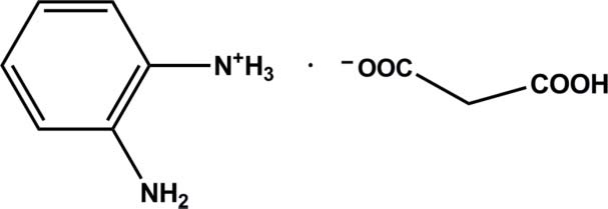


## Experimental

### 

#### Crystal data

C_6_H_9_N_2_^+^·C_3_H_3_O_4_^−^
*M* *_r_* = 212.21
Monoclinic, 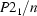

*a* = 12.735 (3) Å
*b* = 5.7448 (11) Å
*c* = 14.429 (3) Å
β = 107.38 (3)°
*V* = 1007.4 (3) Å^3^
*Z* = 4
Mo *K*α radiation
μ = 0.11 mm^−1^
*T* = 298 K
0.30 × 0.25 × 0.15 mm

#### Data collection

Rigaku Mercury2 diffractometer
Absorption correction: multi-scan (*CrystalClear*; Rigaku, 2005 ▶) *T*_min_ = 0.910, *T*_max_ = 1.000
10297 measured reflections
2310 independent reflections
2027 reflections with *I* > 2σ(*I*)
*R*_int_ = 0.026

#### Refinement

*R*[*F* ^2^ > 2σ(*F* ^2^)] = 0.040
*wR*(*F* ^2^) = 0.129
*S* = 1.14
2310 reflections
137 parameters
H-atom parameters constrained
Δρ_max_ = 0.29 e Å^−3^
Δρ_min_ = −0.25 e Å^−3^

### 

Data collection: *CrystalClear* (Rigaku, 2005 ▶); cell refinement: *CrystalClear*; data reduction: *CrystalClear*; program(s) used to solve structure: *SHELXS97* (Sheldrick, 2008 ▶); program(s) used to refine structure: *SHELXL97* (Sheldrick, 2008 ▶); molecular graphics: *SHELXTL* (Sheldrick, 2008 ▶); software used to prepare material for publication: *SHELXTL* .

## Supplementary Material

Crystal structure: contains datablock(s) I, global. DOI: 10.1107/S160053681102544X/rz2617sup1.cif

Structure factors: contains datablock(s) I. DOI: 10.1107/S160053681102544X/rz2617Isup2.hkl

Supplementary material file. DOI: 10.1107/S160053681102544X/rz2617Isup3.cml

Additional supplementary materials:  crystallographic information; 3D view; checkCIF report

## Figures and Tables

**Table 1:** Hydrogen-bond geometry (Å, °)

*D*—H⋯*A*	*D*—H	H⋯*A*	*D*⋯*A*	*D*—H⋯*A*
N1—H1*A*⋯O3^i^	0.89	1.90	2.7865 (17)	171
N1—H1*B*⋯O1^ii^	0.89	1.86	2.7420 (15)	170
N2—H2*A*⋯O3^iii^	0.90	2.21	2.9693 (18)	142
N2—H2*B*⋯O2^iv^	0.90	2.21	3.0988 (18)	169
N1—H1*C*⋯O2	0.89	2.25	2.9019 (15)	130
O4—H4⋯O2	0.82	1.67	2.4616 (15)	161

Symmetry codes: (i) 
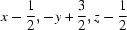
; (ii) 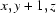; (iii) 
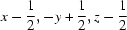
; (iv) 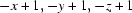.

**Fig. 1.**
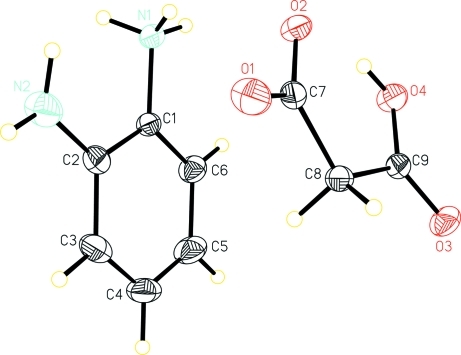
The molecular structure of the title compound with displacement ellipsoids drawn at the 30% probability level.

**Fig. 2.**
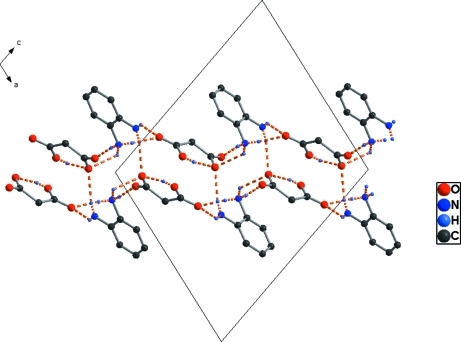
The crystal packing of the title compound viewed along the b axis, showing a double chain formed by intermolecular hydrogen bonds (dashed line). Hydrogen atoms not involved in hydrogen bonding are omitted for clarity.

## supplementary crystallographic information

### Comment

Molecular co-crystals are becoming increasingly important within the pharmaceutical industry as an alternative source of new solid crystalline materials with the potential to provide optimal physical properties whilst retaining the chemical properties of the cocrystal components (Almarsson & Zaworotko, 2004; Blagden *et al.*, 2008; Vishweshwar *et al.*, 2006). Physicochemical properties such as the melting point, stability and solubility of an active pharmaceutical ingredient can be tuned through co-crystal formulation (Kapildev *et al.*, 2011; Schultheiss & Newman, 2009). Co-crystal synthesis often relies on the acid-amide H-bonds interactions. Herein, we report the crystal structure of the title compound, 2-aminoanilinium 2-carboxyacetate.

The asymmetric unit of the title compound is composed of one 2-aminoanilinium cation and one 2-carboxyacetate anion (Fig. 1). The amine N1 atom is protonated and one of the carboxyl groups (C7/O1/O2) is deprotonated. The geometric parameters of the title compound are in the normal range. In the crystal structure, all the amino H atoms are involved in intermolecular N—H···O hydrogen bonding interactions with carboxylic O atoms, linking the ions into double chains parallel to the [101] direction (Table 1; Fig. 2). The conformation of the anion is stabilized by an intramolecular O—H···O hydrogen bond (Table 1).

### Experimental

A mixture of benzene-1,2-diamine (2.0 mmol), malonic acid (2.0 mmol) in distilled water (20 ml) was added into a 50 ml flask and refluxed for 5 hours, then cooled and filtrated. The solution was evaporated slowly in the air. Colourless block crystals suitable for X-ray analysis were obtained after one week.

### Refinement

All H atoms attached to C atoms were fixed geometrically and treated as riding with C-H = 0.93–0.97 Å and with *U*_iso_(H) = 1.2 *U*_eq_(C). The amine and carboxylic H atoms were located in a difference Fourier map and refined freely. In the last stage of the refinement, they were restrained with the H—N1 = 0.90 (2) Å, H—N2 = 0.89 (2) Å and H—O4 = 0.82 (2) Å, and with *U_iso_*(H) = 1.5 *U_eq_*(N1, O4) or 1.2 *U_eq_*(N2).

### Crystal data

**Table:**

C_6_H_9_N_2_^+^·C_3_H_3_O_4_^−^	*F*(000) = 448
*M**_r_* = 212.21	*D*_x_ = 1.399 Mg m^−^^3^
Monoclinic, *P*2_1_/*n*	Mo *K*α radiation, λ = 0.71073 Å
Hall symbol: -P 2yn	Cell parameters from 2310 reflections
*a* = 12.735 (3) Å	θ = 1.9–27.5°
*b* = 5.7448 (11) Å	µ = 0.11 mm^−^^1^
*c* = 14.429 (3) Å	*T* = 298 K
β = 107.38 (3)°	Block, colourless
*V* = 1007.4 (3) Å^3^	0.30 × 0.25 × 0.15 mm
*Z* = 4	

### Data collection

**Table:**

Rigaku Mercury2 diffractometer	2310 independent reflections
Radiation source: fine-focus sealed tube	2027 reflections with *I* > 2σ(*I*)
graphite	*R*_int_ = 0.026
Detector resolution: 13.6612 pixels mm^-1^	θ_max_ = 27.5°, θ_min_ = 1.9°
CCD profile fitting scans	*h* = −16→16
Absorption correction: multi-scan (*CrystalClear*; Rigaku, 2005)	*k* = −7→7
*T*_min_ = 0.910, *T*_max_ = 1.000	*l* = −18→18
10297 measured reflections	

### Refinement

**Table:**

Refinement on *F*^2^	Primary atom site location: structure-invariant direct methods
Least-squares matrix: full	Secondary atom site location: difference Fourier map
*R*[*F*^2^ > 2σ(*F*^2^)] = 0.040	Hydrogen site location: inferred from neighbouring sites
*wR*(*F*^2^) = 0.129	H-atom parameters constrained
*S* = 1.14	*w* = 1/[σ^2^(*F*_o_^2^) + (0.0733*P*)^2^ + 0.1065*P*] where *P* = (*F*_o_^2^ + 2*F*_c_^2^)/3
2310 reflections	(Δ/σ)_max_ < 0.001
137 parameters	Δρ_max_ = 0.29 e Å^−^^3^
0 restraints	Δρ_min_ = −0.25 e Å^−^^3^

### Special details

**Table:**

Geometry. All esds (except the esd in the dihedral angle between two l.s. planes) are estimated using the full covariance matrix. The cell esds are taken into account individually in the estimation of esds in distances, angles and torsion angles; correlations between esds in cell parameters are only used when they are defined by crystal symmetry. An approximate (isotropic) treatment of cell esds is used for estimating esds involving l.s. planes.
Refinement. Refinement of F^2^ against ALL reflections. The weighted R-factor wR and goodness of fit S are based on F^2^, conventional R-factors R are based on F, with F set to zero for negative F^2^. The threshold expression of F^2^ > 2sigma(F^2^) is used only for calculating R-factors(gt) etc. and is not relevant to the choice of reflections for refinement. R-factors based on F^2^ are statistically about twice as large as those based on F, and R- factors based on ALL data will be even larger.

### Fractional atomic coordinates and isotropic or equivalent isotropic displacement parameters (Å^2^)

**Table:**

	*x*	*y*	*z*	*U*_iso_*/*U*_eq_	
O2	0.61965 (7)	0.52957 (17)	0.59590 (7)	0.0380 (3)	
N1	0.58132 (8)	0.80154 (19)	0.41942 (8)	0.0318 (3)	
H1A	0.5336	0.8376	0.3623	0.048*	
H1B	0.5997	0.9297	0.4552	0.048*	
H1C	0.5506	0.6998	0.4500	0.048*	
O4	0.78587 (8)	0.72834 (17)	0.69666 (7)	0.0407 (3)	
H4	0.7231	0.6862	0.6670	0.061*	
O3	0.94464 (8)	0.5435 (2)	0.74114 (7)	0.0458 (3)	
O1	0.62897 (9)	0.1692 (2)	0.54632 (9)	0.0538 (3)	
C1	0.67983 (9)	0.6987 (2)	0.40414 (8)	0.0286 (3)	
C9	0.84796 (9)	0.5516 (2)	0.69323 (8)	0.0303 (3)	
C7	0.67270 (10)	0.3478 (2)	0.58610 (8)	0.0320 (3)	
C8	0.79755 (9)	0.3559 (2)	0.62487 (9)	0.0307 (3)	
H8A	0.8226	0.2101	0.6580	0.037*	
H8B	0.8263	0.3641	0.5698	0.037*	
C6	0.77981 (11)	0.8061 (3)	0.44260 (10)	0.0390 (3)	
H6A	0.7845	0.9435	0.4777	0.047*	
N2	0.56773 (12)	0.3929 (3)	0.31118 (11)	0.0569 (4)	
H2A	0.5601	0.2518	0.2828	0.068*	
H2B	0.5078	0.4222	0.3303	0.068*	
C2	0.66864 (11)	0.4931 (2)	0.35095 (9)	0.0342 (3)	
C3	0.76464 (13)	0.3975 (3)	0.33892 (10)	0.0436 (4)	
H3A	0.7607	0.2596	0.3043	0.052*	
C4	0.86492 (13)	0.5041 (3)	0.37757 (12)	0.0515 (4)	
H4A	0.9277	0.4369	0.3688	0.062*	
C5	0.87378 (12)	0.7085 (3)	0.42893 (12)	0.0513 (4)	
H5A	0.9418	0.7804	0.4542	0.062*	

### Atomic displacement parameters (Å^2^)

**Table:**

	*U*^11^	*U*^22^	*U*^33^	*U*^12^	*U*^13^	*U*^23^
O2	0.0268 (4)	0.0449 (5)	0.0408 (5)	0.0064 (4)	0.0079 (4)	0.0038 (4)
N1	0.0269 (5)	0.0348 (6)	0.0329 (5)	0.0024 (4)	0.0074 (4)	−0.0021 (4)
O4	0.0355 (5)	0.0373 (5)	0.0450 (5)	0.0022 (4)	0.0055 (4)	−0.0112 (4)
O3	0.0282 (5)	0.0568 (6)	0.0444 (6)	−0.0005 (4)	−0.0012 (4)	−0.0073 (5)
O1	0.0412 (6)	0.0616 (7)	0.0592 (7)	−0.0177 (5)	0.0160 (5)	−0.0293 (6)
C1	0.0259 (6)	0.0320 (6)	0.0282 (6)	0.0026 (4)	0.0085 (4)	0.0031 (4)
C9	0.0265 (6)	0.0365 (6)	0.0275 (6)	−0.0006 (5)	0.0075 (4)	0.0004 (5)
C7	0.0277 (6)	0.0421 (7)	0.0262 (5)	−0.0027 (5)	0.0082 (4)	−0.0023 (5)
C8	0.0274 (6)	0.0325 (6)	0.0324 (6)	0.0012 (5)	0.0092 (5)	−0.0031 (5)
C6	0.0319 (6)	0.0423 (7)	0.0414 (7)	−0.0037 (5)	0.0088 (5)	−0.0007 (6)
N2	0.0508 (8)	0.0546 (8)	0.0686 (9)	−0.0159 (6)	0.0229 (7)	−0.0303 (7)
C2	0.0384 (7)	0.0349 (7)	0.0314 (6)	0.0004 (5)	0.0134 (5)	0.0004 (5)
C3	0.0542 (9)	0.0426 (8)	0.0409 (7)	0.0124 (7)	0.0246 (6)	0.0031 (6)
C4	0.0425 (8)	0.0685 (11)	0.0513 (9)	0.0203 (7)	0.0259 (7)	0.0154 (8)
C5	0.0279 (7)	0.0690 (11)	0.0571 (9)	−0.0014 (7)	0.0128 (6)	0.0069 (8)

### Geometric parameters (Å, °)

**Table:**

O2—C7	1.2742 (16)	C8—H8A	0.9700
N1—C1	1.4618 (15)	C8—H8B	0.9700
N1—H1A	0.8900	C6—C5	1.388 (2)
N1—H1B	0.8900	C6—H6A	0.9300
N1—H1C	0.8900	N2—C2	1.3679 (19)
O4—C9	1.2969 (16)	N2—H2A	0.9003
O4—H4	0.8221	N2—H2B	0.9008
O3—C9	1.2195 (15)	C2—C3	1.3978 (19)
O1—C7	1.2253 (17)	C3—C4	1.375 (2)
C1—C6	1.3737 (18)	C3—H3A	0.9300
C1—C2	1.3924 (18)	C4—C5	1.375 (3)
C9—C8	1.5069 (17)	C4—H4A	0.9300
C7—C8	1.5205 (17)	C5—H5A	0.9300
			
C1—N1—H1A	109.5	C7—C8—H8B	108.0
C1—N1—H1B	109.5	H8A—C8—H8B	107.2
H1A—N1—H1B	109.5	C1—C6—C5	119.72 (14)
C1—N1—H1C	109.5	C1—C6—H6A	120.1
H1A—N1—H1C	109.5	C5—C6—H6A	120.1
H1B—N1—H1C	109.5	C2—N2—H2A	122.0
C9—O4—H4	105.0	C2—N2—H2B	124.6
C6—C1—C2	122.23 (12)	H2A—N2—H2B	108.7
C6—C1—N1	119.42 (12)	N2—C2—C1	121.12 (12)
C2—C1—N1	118.34 (11)	N2—C2—C3	121.96 (13)
O3—C9—O4	122.15 (12)	C1—C2—C3	116.91 (13)
O3—C9—C8	120.19 (12)	C4—C3—C2	121.01 (14)
O4—C9—C8	117.62 (10)	C4—C3—H3A	119.5
O1—C7—O2	123.81 (12)	C2—C3—H3A	119.5
O1—C7—C8	118.48 (12)	C5—C4—C3	121.04 (13)
O2—C7—C8	117.71 (11)	C5—C4—H4A	119.5
C9—C8—C7	117.25 (10)	C3—C4—H4A	119.5
C9—C8—H8A	108.0	C4—C5—C6	119.08 (14)
C7—C8—H8A	108.0	C4—C5—H5A	120.5
C9—C8—H8B	108.0	C6—C5—H5A	120.5
			
O3—C9—C8—C7	165.75 (12)	C6—C1—C2—C3	0.67 (19)
O4—C9—C8—C7	−16.10 (16)	N1—C1—C2—C3	−179.06 (11)
O1—C7—C8—C9	−165.98 (12)	N2—C2—C3—C4	178.41 (14)
O2—C7—C8—C9	14.93 (16)	C1—C2—C3—C4	−0.5 (2)
C2—C1—C6—C5	−0.2 (2)	C2—C3—C4—C5	−0.2 (2)
N1—C1—C6—C5	179.56 (12)	C3—C4—C5—C6	0.8 (2)
C6—C1—C2—N2	−178.23 (14)	C1—C6—C5—C4	−0.6 (2)
N1—C1—C2—N2	2.05 (19)		

### Hydrogen-bond geometry (Å, °)

**Table:**

*D*—H···*A*	*D*—H	H···*A*	*D*···*A*	*D*—H···*A*
N1—H1A···O3^i^	0.89	1.90	2.7865 (17)	171
N1—H1B···O1^ii^	0.89	1.86	2.7420 (15)	170
N2—H2A···O3^iii^	0.90	2.21	2.9693 (18)	142
N2—H2B···O2^iv^	0.90	2.21	3.0988 (18)	169
N1—H1C···O2	0.89	2.25	2.9019 (15)	130
O4—H4···O2	0.82	1.67	2.4616 (15)	161

Symmetry codes: (i) *x*−1/2, −*y*+3/2, *z*−1/2; (ii) *x*, *y*+1, *z*; (iii) *x*−1/2, −*y*+1/2, *z*−1/2; (iv) −*x*+1, −*y*+1, −*z*+1.
